# Combining segments 9 and 10 in DNA and recombinant protein vaccines conferred superior protection against tilapia lake virus in hybrid red tilapia (*oreochromis* sp.) compared to single segment vaccines

**DOI:** 10.3389/fimmu.2022.935480

**Published:** 2022-07-25

**Authors:** Pitakthai Chamtim, Eukote Suwan, Ha Thanh Dong, Soranuth Sirisuay, Nontawith Areechon, Eakapol Wangkahart, Ikuo Hirono, Rapeepat Mavichak, Sasimanas Unajak

**Affiliations:** ^1^ Department of Biochemistry, Faculty of Science, Kasetsart University, Bangkok, Thailand; ^2^ Faculty of Veterinary Technology, Kasetsart University, Bangkok, Thailand; ^3^ Aquaculture and Aquatic Resources Management Program, Department of Food, Agriculture and Bioresources (AARM/FAB), School of Environment, Resources and Development, Asian Institute of Technology, Pathum Thani, Thailand; ^4^ Department of Aquaculture, Faculty of Fisheries, Kasetsart University, Bangkok, Thailand; ^5^ Division of Fisheries, Department of Agricultural Technology, Faculty of Technology, Mahasarakham University, Maha Sarakham, Thailand; ^6^ Graduate School of Marine Science and Technology, Tokyo University of Marine Science and Technology, Tokyo, Japan; ^7^ Molecular Biology Research Department, Charoen Pokphand Foods Public Co., Ltd., Aquatic Animal Health Research Center, Samut Sakhon, Thailand

**Keywords:** DNA vaccine, TiLV, tilapia (fish), recombinant protein vaccine, TiLV ORF10, TiLV ORF9

## Abstract

Tilapia lake virus (TiLV) now affects Nile tilapia culture worldwide, with no available commercial vaccine for disease prevention. DNA and recombinant protein-based vaccines were developed and tested following viral isolation and characterization. The viral strain isolated from diseased hybrid red tilapia (*Oreochromis* sp.) shared high levels of morphological and genomic similarity (95.49-99.52%) with other TiLV isolates in the GenBank database. TiLV segment 9 (Tis9) and segment 10 (Tis10) DNA vaccines (pcDNA-Tis9 and pcDNA-Tis10) and recombinant protein vaccines (Tis9 and Tis10) were prepared and tested for their efficacy in juvenile hybrid red tilapia. Fish were immunized with either single vaccines (pcDNA-Tis9, pcDNA-Tis10, Tis9 and Tis10) or combined vaccines (pcDNA-Tis9 + pcDNA-Tis10 and Tis9 + Tis10) by intramuscular injection and intraperitoneal injection for DNA and protein vaccines, respectively. Negative controls were injected with PBS or a naked pcDNA3.1 vector in the same manner. An experimental challenge with TiLV was carried out at 4 weeks post-vaccination (wpv) by intraperitoneal injection with a dose of 1 × 10^5^ TCID_50_ per fish. Relative percent survival (RPS) ranged from 16.67 ± 00.00 to 61.11 ± 9.62%. The Tis10 and pcDNA-Tis10 vaccines conferred better protection compared to Tis9 and pcDNA-Tis9. Highest levels of protection were observed in pcDNA-Tis9 + pcDNA-Tis10 (61.11 ± 9.62%) and Tis9 + Tis10 (55.56 ± 9.62%) groups. Specific antibody was detected in all vaccinated groups at 1-4 wpv by Dot Blot method, with the highest integrated density at 2 and 3 wpv. *In silico* analysis of Tis9 and Tis10 revealed a number of B-cell epitopes in their coil structure, possibly reflecting their immunogenicity. Findings suggested that the combination of Tis9 and Tis10 in DNA and recombinant protein vaccine showed high efficacy for the prevention of TiLV disease in hybrid red tilapia.

## Introduction

Tilapia lake virus (*Tilapia tilapinevirus*, TiLV), a member of the family *Amnoonviridae* and the order *Articulavirales*, is a significant cause of infection that affects mass die-offs in tilapia culture system all around the world. Specifically, rapid mortality of both black and red tilapia fingerlings was found in TiLV infection (1–3). Practical methods such as disinfectants and farm management do not prevent and control the spread of TiLV. Effective and practical alternative methods of disease prevention and control such as vaccination should be developed to solve the problem.

Vaccines are a promising method for disease prevention and control but no commercial vaccines against TiLV disease are currently available. Thus, inactivated TiLV and recombinant VP20 (TiLV segment 8) were developed as injectable vaccines (4–6). In addition to vaccine efficacy, which varies depending on preparation method and adjuvant (5–7), the vaccine’s safety profiles must also be considered in vaccine selection. Therefore, subunit recombinant protein and DNA vaccines are more interesting and in demand. Modern subunit recombinant protein and DNA vaccines are used in humans, animals and fish including TiLV vaccines for disease control. Several modern fish vaccines have been commercialized. Modern vaccines are designed by a reverse vaccinology approach using a pathogen’s genome or proteome profile.

The whole genome sequence of the TiLV consists of a negative-sense single-stranded RNA with 10,323 bases arranged onto 10 viral genome segments (8). All viral genome segments have low similarity to known functional proteins. TiLV genome segment 1 putative protein shows weak homology to the RNA-dependent polymerase PB1 subunit of influenza C virus (8) whereas other proteins are classified as hypothetical for which their function is unknown. Recently, a combinatorial sequence- and structure-based analysis on the functional proteome of TiLV uncovered transmembrane helix regions in encoded TiLV segments 1, 3 and 9 proteins (7), while *in silico* analysis of the TiLV genome found nucleolar and nuclear localization signals in segment 10 (9).

Although a recent study on VP20 showed maximum protection of about 71.8% RPS in the group of fish primarily immunized with VP20 DNA vaccine followed by booster with recombinant VP20 protein in adjuvants (6). This DNA prime-protein boost strategy also required booster immunization (3^rd^ week) for small tilapia fish to achieve promising protection. However, multiple administration of vaccine by injection to a large number of small fishes has some drawbacks, such as increased stress and adverse effects on the fish. In addition, there are not practical and cost-effective by increasing labor cost and time required for vaccine administration at the large-scale hatchery (10–12).

Therefore, the aim of this study is to develop the effective injectable vaccines against TiLV infection by only single vaccination. Interestingly, our rational vaccine design for TiLV protection regarding to combine multiple antigens into a vaccine. We expected these combined vaccines might provide stronger and prolong immune protection against TiLV compared with the single vaccine. Considerably, combined vaccines containing more than one antigen have the advantage to stimulate the production of different antibodies which specific to various antigens, and those antibodies are sufficient to protect against viral infection (13–15).

Herein, recombinant proteins from 8 out of 10 segments of the TiLV genome were successfully produced and purified, excluding segments 5 and 6. Considering the wide application of TiLV vaccine against different strains of TiLV virus, conserved and high yield recombinant TiLV proteins should be tested for their vaccine efficacy against TiLV. Therefore, the recombinant protein and DNA vaccines used in this study were prepared from TiLV segment 9 (Tis9) and 10 (Tis10). Fish were vaccinated with either single (pcDNA-Tis9, pcDNA-Tis10, Tis9 and Tis10) or combined vaccines (pcDNA-Tis9 + pcDNA-Tis10 and Tis9 + Tis10), and were then evaluated the immune response. Vaccine efficacy and the related epitopes of Tis9 and Tis10 were affirmed by increasing specific antibodies and *in silico* analysis. Our work provided the development of the effective vaccine to protect against TiLV infection.Materials and methods

### Experimental fish

During TiLV outbreaks in 2018, red tilapia (*Oreochromis* spp.) with clinical symptoms or abnormal behaviors were collected from floating cages of local tilapia farms in Thailand. All infected fish demonstrated clinical signs and the internal tissues (i.e., brain, liver and spleen) were collected and preserved in RNAlater™ for TiLV detection, while the remaining tissues were pooled in Leibovitz (L-15) medium (Sigma) and stored at -80°C for TiLV isolation. This experiment on animals complied with the ethical standards set by National Research Council of Thailand (NRCT).

### RNA extraction and cDNA preparation

Total RNA was extracted from the brain, liver and spleen using TRIzol™ reagent (Invitrogen, USA), and processed for RNA extraction according to the manufacturer’s instruction. Concentration and purity of RNAs were quantified using a Nanodrop 2000 Spectrophotometer (Thermo Scientific, USA) and gel electrophoresis.

For cDNA synthesis, the RNA in each sample was reverse transcribed using a Viva 2-steps RT-PCR kit (Vivantis, Malaysia) in a 20 µL reaction. One microgram of total RNA was mixed with 4 µM oligo dT primer and 1 mM dNTP and the final volume was adjusted to 10 µL with nuclease-free water. The reaction was incubated at 65°C for 5 min followed by 4°C for 2 min. Then, 10 µL of cDNA synthesis mixture was added to the reaction, incubated at 42°C for 60 min, and the reaction was stopped by heat inactivation at 85°C for 5 min.

### TiLV detection

For TiLV detection, cDNA was used as the template for the PCR reaction with SpecificTiLV-F and SpecificTiLV-R, as shown in **Supplementary Table 1**. Each PCR reaction was conducted in a volume of 20 µL containing 2 µL of cDNA template, 0.5 µM of forward and reverse primers, 2 µL of 10× Dream *Taq* buffer, 0.2 mM of each dNTP and 0.2 µL of 1 U *Taq* DNA polymerase. The PCR condition consisted of denaturation at 95°C for 5 min, followed by 35 cycles of 95°C for 30 s, 55°C for 30 s, and 72°C 1 min, and final elongation at 72°C for 10 min. PCR products were electrophoresed on 1% agarose gel and visualized by LAS500 (GE).

#### TiLV propagation in E-11 and TK-1 cell lines

TiLV was isolated from the internal tissues of the collected fish. Briefly, the tissues were homogenized in L-15 medium (L-15 Leibovitz medium, Sigma) and then centrifuged at 3,000 ×g for 10 min at 4°C. The suspension was collected and filtered through a 0.22 µm membrane filter. The filtrate was collected and inoculated into E-11 cells [continuous cell line from snakehead fish (*Ophicephalus striatus*)] and TK-1 cells (a continuous cell line from °the kidney of °hybrid *Tilapia mossambica* and *T. nilotica*). The cells were maintained in L-15 medium supplemented with 10% fetal bovine serum (FBS) at 27°C without CO_2_ for 10 days. The cytopathic effects (CPEs) were observed daily. At the end of the experiment, the culture media were harvested using centrifugation at 3,000 ×g for 10 min at 4°C and stored at -80°C for further use.

### TiLV isolation and purification using sucrose gradient fractionation

For TiLV purification, the culture media from TiLV inoculated TK1 cells were centrifuged for 10 min at 1,400 × g. The supernatant was collected and washed with 30% (wt/vol) sucrose–Tris-EDTA (TE) buffer and centrifuged. After discarding the supernatant, the pellets were suspended in TE buffer and overlaid onto 3 mL of a gradient layer of 70, 60, 50, 40, 30, 20 and 10% (wt/vol) sucrose–TE buffer. TiLV virions were collected from each gradient by ultracentrifugation (UC), resuspended in 1 mL of TE buffer and preserved at -80°C for subsequent analysis.

### Transmission electron microscopy analysis

To examine the morphology, 100 µL of purified TiLV was diluted with phosphate buffered saline (PBS) at a ratio of 1:10. Five microliters of the diluent were mixed with 3% (vol./vol.) phosphotungstic acid (PTA) (kindly provided by Prof. Porntippa Lekchareonsuk, Kasetsart University). Then, 10 µL of the mixture was dropped on a thin copper grid for 10 min and examined at 80 kilovolts (kV) using a Hitachi HT7700 Transmission Electron Microscope (TEM) (Hitachi, Germany) at the Scientific Equipment and Research Division, Kasetsart University, Bangkok, Thailand.

### Amplification of TiLV genome segments

The cDNA of the TiLV genome was used as a template for amplifying 10 segments of the viral genome. Specific primers were designed according to the coding sequences (CDS) of the reference TiLV genome available in the GenBank database (primers: Tis1-F and R, Tis2-F and R, Tis3-F and R, Tis4-F and R, Tis5-F and R, Tis6-F and R, Tis7-F and R, Tis8-F and R Tis9-F and R and Tis10-F and R, as shown in **Supplementary Table 1**).

PCR amplification of Tis was achieved using the cDNA of the viral genome with denaturation at 95°C for 5 min, followed by 35 cycles at 95°C for 30 s, 56°C for 30 s and 72°C for 1-3 min (depending on the length of the target genes), with a final elongation at 72°C for 5 min. The PCR products were purified using a GeneJET purification kit and cloned to the pGEM-T^®^ Easy vector (Promega), generating recombinant cloning plasmids designated as pTis-1, pTis-2, pTis-3, pTis-4, pTis-5, pTis-6, pTis-7, pTis-8, pTis-9 and pTis-10 for TiLV segments 1-10, respectively. Authentic sequences were verified by DNA sequencing and BLASTn analysis against the NCBI nucleotide database. TiLV genome sequences were deposited in the NCBI GenBank as accession numbers: OL469273 to OL469282 (unpublished data and accessed on 25 December 2021).

### TiLV sequence analysis

The open reading frame (ORF)-encoded protein prediction as well as physicochemical properties of Tis 1-Tis 10 were analyzed using ORF Finder (16) and ProtParam online server (http://us.expasy.org/tools/protparam.html) (17), respectively. Sequences of the coding region Tis 1-Tis 10 were compared with the available sequences of the original TiLV isolate from Israel (GenBank accession numbers: KU751814 to KU751823) using Nucleotide BLAST (blastn) in the GenBank database (NCBI). The results of the sequence similarity search were presented as pairwise alignments.

### Recombinant Tis protein vaccine preparation

All verified recombinant cloning plasmids (pTis-1 to pTis-10, with the exception of pTis-5 and pTis-6) and pET32-a vector (Novagen), were digested with the corresponded restriction enzymes and ligated to construct the recombinant protein expression plasmids. The recombinant TiLV expression plasmids were designated as pET32-Tis -1 to 4 and pET32-Tis-7 to 10, respectively. The constructed recombinant expression plasmids were then transformed into *E. coli* BL21 (DE3) pLySs and positive colonies were grown on 50 mg. mL^-1^ of ampicillin (1:2,000) and 34 mg. mL^-1^ of chloramphenicol (1:1,000) containing Luria Bertani (LB) agar plates. Post-induction temperatures and times (15°C for 16 hr 30°C for 6 hr and 37°C for 4 hr) at small scale expression were varied to determine the optimal expression conditions for Tis proteins.

For large-scale expression, the cells were cultured in LB broth and incubated at 37°C with 220 rpm shaking until OD_600_ reached 0.6. Then, 1 mM of isopropyl-β-D-thiogalactoside (IPTG) was added, with continued incubation at 37°C and shaking at 220 rpm for 4 hr. The cells were harvested by centrifuging at 10,000 ×g, 4°C for 20 min. The cells were then resuspended in lysis buffer (50 mM Tris pH 8.0, 500 mM NaCl, 10 mM imidazole, 0.1% Triton X-100, 2mM PMSF and 1 mg/mL lysozyme) 3 mL per 1 g of cell pellet and lysed by sonication. The obtained cell lysate was then centrifuged at 10,000 x g, 4°C for 20 min to collect the supernatant. The expressed Tis proteins were purified using Ni-NTA (nickel-nitrilotriacetic acid) agarose beads (Qiagen). The obtained supernatant was incubated with a Ni-NTA affinity column at 4 °C for 1 hr, and then washed with washing buffer (20 mM Tris pH 8.0, 300 mM NaCl and 20 mM imidazole) to effectively remove nonspecific bindings before being eluted with elution buffer (20 mM Tris pH 8.0, 300 mM NaCl and 250 mM imidazole). To determine the insoluble protein, the pellet was resuspended in lysis buffer containing 4 M urea, lysed by sonication and then centrifuged (10,000 x g, 4°C for 20 min) to collect the clear supernatant, which was subjected to purification using a Ni-NTA affinity column. The column was then washed with washing buffer and eluted with 4 M urea elution buffer (20 mM Tris pH 8.0, 300 mM NaCl, 250 mM imidazole and 4 M urea). The purified Tis-proteins were concentrated using Vivaspin^®^ 20 mL centrifugal concentrators (10,000 Da molecular weight cut-off). These Tis-proteins were examined by sodium dodecyl sulfate polyacrylamide gel electrophoresis (SDS-PAGE).

### Tis9 and Tis10 DNA vaccine preparation

To prepare Tis9 and Tis10 DNA vaccine, pcDNA3.1(+) plasmid was used as the vector to construct pcDNA-Tis9 or pcDNA -Tis10 plasmids. Briefly, Tis9 and Tis10 genes were amplified by PCR with TiLV-S9 and TiLV-S10 primers, (**Supplementary Table 1**). The amplicons were digested with the corresponding restriction enzymes and then integrated into the pcDNA3.1(+) plasmid at the corresponded sites. The authenticity of Tis9 and Tis10 sequences in pcDNA3.1(+) were verified by nucleotide sequencing (Macrogen, Korea). The plasmids harboring Tis9 and Tis10 DNA vaccine and pcDNA3.1(+) plasmid were extracted from *E. coli* DH5α using cesium chloride (CsCl) gradient ultracentrifugation (18) and suspended in TE buffer at pH 8.0 for immunization.

### *In vivo* efficacy analysis of Tis9 and Tis10 recombinant protein and DNA vaccines on TiLV infection

Pathogen-free red tilapia (*Oreochromis* spp.) were acclimatized for 3 weeks and fed with a commercial diet daily. The water was partially replaced every day and water quality as temperature, dissolved oxygen, pH, ammonia and nitrite were monitored every other day. Before vaccination, the brain, liver and kidney of the fish were sampled and screened for TiLV infection using PCR analysis.

To explore the efficacy of the developed vaccines, 600 healthy fish (35.0 ± 3.0 g) were equally divided into 8 groups as 1) PBS (negative control of protein vaccine), 2) Tis9 protein vaccine, 3) Tis10 protein vaccine, 4) a mixture of Tis9 and Tis10 proteins (1:1) vaccine, 5) pcDNA3.1(+) plasmid (negative control of DNA vaccine), 6) pcDNA-Tis9, 7) pcDNA-Tis10 and 8) a mixture of pcDNA-Tis9 and pcDNA-Tis10 (1:1). For protein vaccination (groups 2, 3 and 4), purified Tis proteins were diluted in 1x PBS and mixed with Montanide ISA 763 (Seppic, France) before intraperitoneal injection with 200 µg of protein per fish. For DNA vaccination (groups 6, 7 and 8), 5 µg of DNA vaccine plasmids were applied to the fish through intramuscular injection. All treatments were conducted in triplicate and the fish were maintained in tanks containing 30 L of aerated water at 28 ± 2°C and fed twice per day with commercial pellet feed. To evaluate the antibody response, blood samples of three fish from each treatment group were collected and the serum was separated to detect specific antibodies by dot-blot immunoassay every week after vaccination. At four weeks post-vaccination, 10 experimental fish per replicate were randomly selected and challenged with TiLV diluted at 1 × 10^5^ TCID_50_ mL^-1^ (kindly provided by Dr. Ha Thanh Dong) by intraperitoneal injection. Rates of mortality and moribund fish were observed daily for 4 weeks after the challenge. Dead fish were randomly selected to identify TiLV infection from the brain, liver and spleen. The cumulative mortality and the relative percent of survival (RPS) were calculated as previously reported (19) using the following: RPS (%) = [1- (% mortality of the vaccine-treated group/% mortality of the PBS-control group)] x 100.

### Specific antibody determination

Specific antibody responses for Tis recombinant protein and DNA vaccination were detected by dot-blot immunoassay as described previously with some modifications (18), using the Minifold^®^ I dot blot system (GE Healthcare, Germany). Briefly, 20 µL purified Tis9 and Tis10 proteins were dotted on a nitrocellulose membrane, incubated for 15 min and blocked with 1% BSA in Tris-buffered saline with Tween (TBST) for 30 min. Then, 10 µL of the fish sera collected from the different treatment groups were added and incubated for 1 hr. The membrane was probed with a primary antibody (anti-tilapia IgM at 1:5,000) for 2 hr, followed by washing three times with TBST buffer and incubating with an anti-mouse IgG HRP-linked Ab (1:10,000) for 1 hr. Subsequently, signal development was performed to the substrate reagent (Immobilon^®^ Forte, USA) and detected with a ChemiDoc™ Imaging System (Bio-Rad). The integrated density of each dot was analyzed by Image Lab Software version 6.1 (Bio-Rad) and normalized with PBS and pcDNA3.1(+) (negative control group).

### *In silico* determination of Tis protein immunogenicity

Secondary structures of Tis9 and Tis10 proteins were predicted using the online tool RaptorX Property Server (http://raptorx.uchicago.edu/StructurePropertyPred/predict/), which included secondary structure (SS) and solvent accessibility (ACC). This server uses a machine learning model called DeepCNF (Deep Convolutional Neural Fields) to continuously compute the secondary structure and solvent accessibility (20). The tertiary or three-dimensional (3D) structures of Tis9 and Tis10 proteins were rendered by the homology modeling tool I-TASSER (Iterative Threading Assembly Refinement) server (https://zhanglab.ccmb.med.Umich.Edu/I-TASSER/) (21). The derived I-TASSER models with the highest confidence score (C-score) in the PDB files were selected for refinement analysis using GalaxyRefine Server (http://galaxy.seoklab.org/cgi-bin/submit.cgi?type=REFINE) (22). The final refined 3D models were analyzed using PROCHECK v.3.5 to generate a Ramachandran plot that determined the overall quality of the tertiary structures (23). The immunogenicities of Tis9 and Tis10 proteins were evaluated by the prediction of the antigenicity, linear and conformational B cell epitopes. The antigenicity was analyzed with °the VaxiJen v2.0 server °at a 0.4% threshold for the virus model (24). Linear and conformational B cell epitopes of the validated 3D structure model were predicted by the ElliPro Server (http://tools.iedb.org/ellipro) (25), with epitope prediction parameters set to minimum score ≥ 0.6 and maximum distance ≥ 5 angstroms. Linear and conformational B cell epitopes were mapped to the predicted 3D structure of Tis9 and Tis10 proteins visualized *via* visual molecular dynamics (VMD) v.1.9 (26).

### Statistical analysis

Statistical analysis was performed using GraphPad Prism 7 (GraphPad Software, Inc. San Diego, CA, USA). Trial data were presented as mean ± standard deviation (SD). Differences among the groups were analyzed using one-way analysis of variance (one-way ANOVA). Multiple comparison was performed by Turkey’s *post hoc* test and considered significant at p < 0.05.

## Results

### Propagation and isolation of TiLV from TK-1

**Figure 1:**
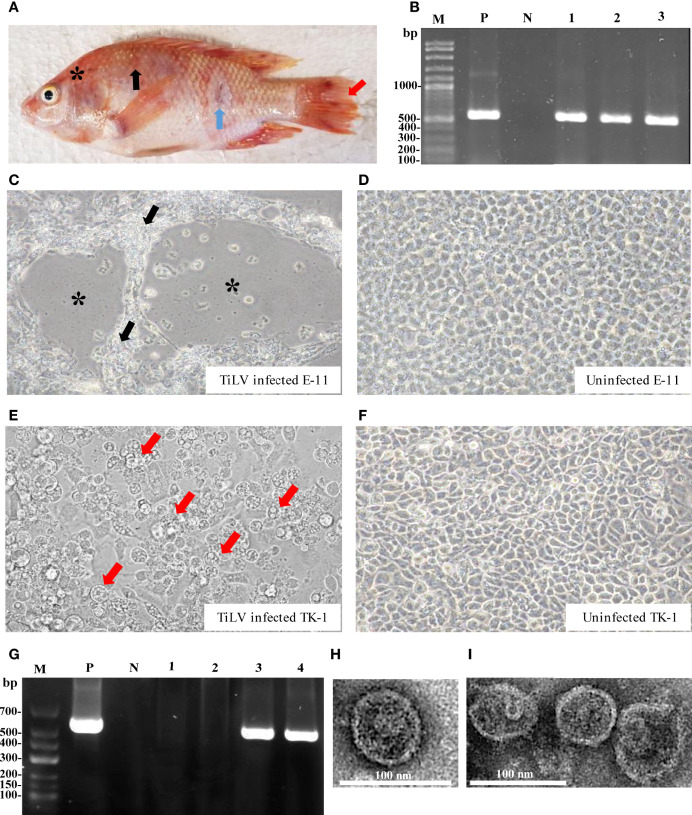
TiLV detection of infected tilapia organs, infected cell cultures and transmission electron microscope (TEM) analyses. **(A)** Clinical signs of infected fish include skin redness and congestion around the eye and head (asterisk), severe skin hemorrhage (black arrow), skin erosion (blue arrow) and fin rot (red arrow). **(B)** Infected fish were screened for TiLV infection from different organs using RT-PCR and specific PCR products approximately 500 bps were observed. Lane M: DNA marker; Lane P: positive control; Lane N: negative control; Lanes 1-3: brain, liver and spleen tissues from infected fish, respectively. **(C)** E-11 infected cells revealed CPEs with plaque formation (asterisk) and syncytial formation (black arrow) at 7 days post infection (dpi). **(D)** Uninfected E-11 cells. **(E)** TK-1 infected cells revealed CPEs with cell swelling, vacuolated appearance (red arrow) followed by cell detachment at 7 dpi. **(F)** Uninfected TK-1 cells. **(G)** Detection of TiLV from E-11 and TK-1 infected cells by RT-PCR. Lane M: DNA marker; Lane P: positive control; Lane N: negative control; Lanes 1-2: uninfected E-11 and TK-1, respectively; Lanes 3-4: infected E-11 and TK-1 cells, respectively. **(H, I)** TEM micrograph of the purified TiLV from infected TK-1 culture supernatant that showed isolated virion **(H)**, and aggregated virions in small group **(I)**.

During 2018, Nile tilapia culture was severely impacted by TiLV (27). Many fish showed clinical signs of skin redness and congestion around the eye and head, with severe skin hemorrhage and erosion (**Figure 1A**). RT-PCR analyses showed that the fish were heavily infected by TiLV in different organs (**Figure 1B**).

*In vitro* propagation of TiLV was performed by inoculating homogenate from internal tissues of TiLV infected tilapia into two different cell lines including E-11 and TK-1 cells. The CPEs in E-11 cells were characterized by cell shrinkage and aggregation, plaque formation and syncytial formation, which became visible 5 to 7 days post-inoculation (**Figure 1C**), and cell detachment progressed within 8 days post-inoculation. In TK-1 cells, CPEs were clearly observed within 5 days post-inoculation, with the appearance of granulated cells or intracytoplasmic vacuolation, cell swelling and rounding, leading to disintegration of the cell monolayer at 7 days post-inoculation (**Figure 1E**). The control E-11 and TK-1 cells (mock-group) did not show any CPEs (**Figures 1D, F**). Infection and propagation of TiLV in E-11 and TK-1 cells were verified by RT-PCR, showing positive for TiLV in E-11 and TK-1 cells only (**Figure 1G**).

Subsequently, purification of TiLV from TK-1 cell cultures was achieved by sucrose gradient fractionation, and the purified cultures were verified by size, shape and integrity through TEM analysis. As shown in **Figures 1H, I**, the purified virus revealed rounded-shape viral particles with average diameters of between 75 and 80 nm. A smooth thick envelope of the virus was also observed.

### Molecular characterizations of TiLV genome segments

The coding DNA sequence (CDS) and encoded amino acids of the TiLV genome segments, together with the biochemical properties of the predicted protein are presented in **Table 1**. Sequencing and pairwise CDS alignment analyses of TiLV segments 1-10 from fish revealed 95.49 to 98.10% nucleotide identity and 95.92 to 99.52% amino acid identity to the corresponding coding region of nucleotide sequences of the original TiLV isolate (NCBI accession no. KU751814 - KU751823) from Israel (4), as shown in **Table 2**.

**Table 1:** ORF-encoded protein prediction and biochemical properties of TiLV segments 1-10 (Tis1 - Tis10) proteins.

TiLV genesegment	GenBank accession no.^a^	Gene segment length (bp)	Predicted protein name	Predicted protein length (aa)	MW (kDa)	pI	Hydropathicity	Aliphatic index
Segment 1	OL469273	1560	Tis1	519	57.109	8.36	-0.150	84.95
Segment 2^b^	OL469274	840	Tis2	279	31.492	9.39	-0.451	76.95
Segment 3	OL469275	1260	Tis3	419	47.689	7.56	-0.422	81.24
Segment 4	OL469276	1076	Tis4	356	38.577	9.13	-0.140	85.81
Segment 5	OL469277	1032	Tis5	343	38.05	8.59	0.262	101.69
Segment 6	OL469278	936	Tis6	311	35.68	8.79	-0.316	79.26
Segment 7	OL469279	588	Tis7	195	21.853	9.67	-0.310	76.97
Segment 8	OL469280	525	Tis8	174	19.536	9.27	-0.167	100.34
Segment 9	OL469281	360	Tis9	119	13.539	6.04	-0.143	82.61
Segment 10	OL469282	342	Tis10	113	12.803	4.63	-1.241	50.09

a Unpublished data (accessed on 25 December 2021).

b The PCR product of TiLV segment 2 was amplified with primers Tis-2-F and Tis-2-R (**Supplementary Table 1**) to obtain the Tis-2 protein, which had 279 amino acids and was translated from the second start codon (position 184).

**Table 2:** Nucleotide and amino acid identity of coding region of TiLV segments 1-10 in this study and the original TiLV isolate from Israel.

TiLV segment	GenBank accession no. (this study)	Nucleotide identity (%)	Amino acid identity (%)
Segment 1	OL469273	96.22	98.84
Segment 2	OL469274	97.31	99.27
Segment 3	OL469275	96.67	99.52
Segment 4	OL469276	96.36	98.31
Segment 5	OL469277	96.12	95.92
Segment 6	OL469278	95.49	96.58
Segment 7	OL469279	96.77	99.49
Segment 8	OL469280	98.10	98.21
Segment 9	OL469281	97.76	97.46
Segment 10	OL469282	97.95	97.35

### Expression and purification of Tis9 and Tis10 recombinant proteins

**Figure 2:**
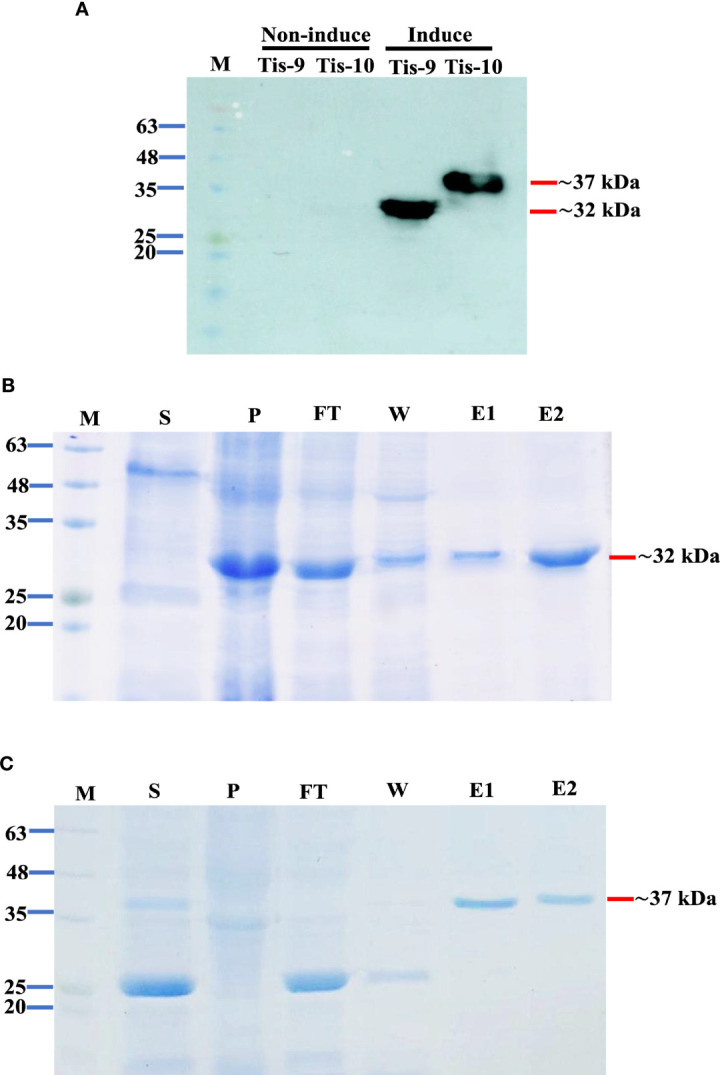
Expression and purification of Tis9 and Tis10 proteins. **(A)** Western blot analysis of both proteins from the *E. coli* expression system. Tis9 and Tis10 proteins expressed approximately 32 kDa and 37 kDa, respectively (Lane M: prestained protein marker). **(B, C)** SDS-PAGE analysis of **(B)** Tis9 and **(C)** Tis10 proteins purification with coomassie blue staining. (Lane M: prestained protein marker; Lane S: supernatant; Lane P: precipitation of the *E. coli* was induced; Lane F: unbound flowthrough; Lane W: wash fraction; Lanes: E1-E2 = elute fractions.

Small scale expression with several post-induction temperatures and times including 15°C for 16 hr, 30°C for 6 hr and 37°C for 4 hr was optimized to develop a recombinant protein vaccine to control TiLV infection. The recombinant pET32a (+) plasmids containing Tis genes (except Tis-5 and Tis-6) were constructed and successfully expressed all Tis proteins in all expression conditions. Notably, Tis9 and Tis10 proteins were more highly expressed than the others (data not shown). The highest recombinant protein expressions; Tis9 (32 kDa) and Tis10 (37 kDa), were chosen for large-scale production (**Figure 2A**) and further analyzed as candidate vaccines to control TiLV infection. The purity of both proteins after affinity chromatography purification was assessed by SDS-PAGE analysis, and results revealed high integrity suitable for use as a vaccine (**Figures 2B, C**).

### Efficacy of Tis9 and Tis10 recombinant proteins and DNA vaccines

Two vaccine systems; Tis9 and Tis10 recombinant protein vaccine and DNA vaccine, were used to verify efficacy in tilapia. At 4 weeks after vaccination, fish in each group were challenged by intraperitoneal injection with 1 x 10^5^ TCID_50_ TiLV. The moribund fish showed clinical signs of TiLV infection, such as skin redness and congestion around the eye and head, pale skin with hemorrhage and erosion, and fin rot. These moribund fish were collected, and TiLV were screened, showing that they were infected with TiLV virus (**Figure S1**). The first mortality was observed on day 7 or 8 post-challenge in PBS, Tis9, Tis10, pcDNA-Tis10 and pcDNA-Tis9 + Tis10 groups. After that, the challenged fish in pcDNA3.1 and Tis9 + Tis10 recombinant protein vaccine groups began to die on day 10 post-challenge, followed by the pcDNA-Tis9 group on day 14.

**Figure 3:**
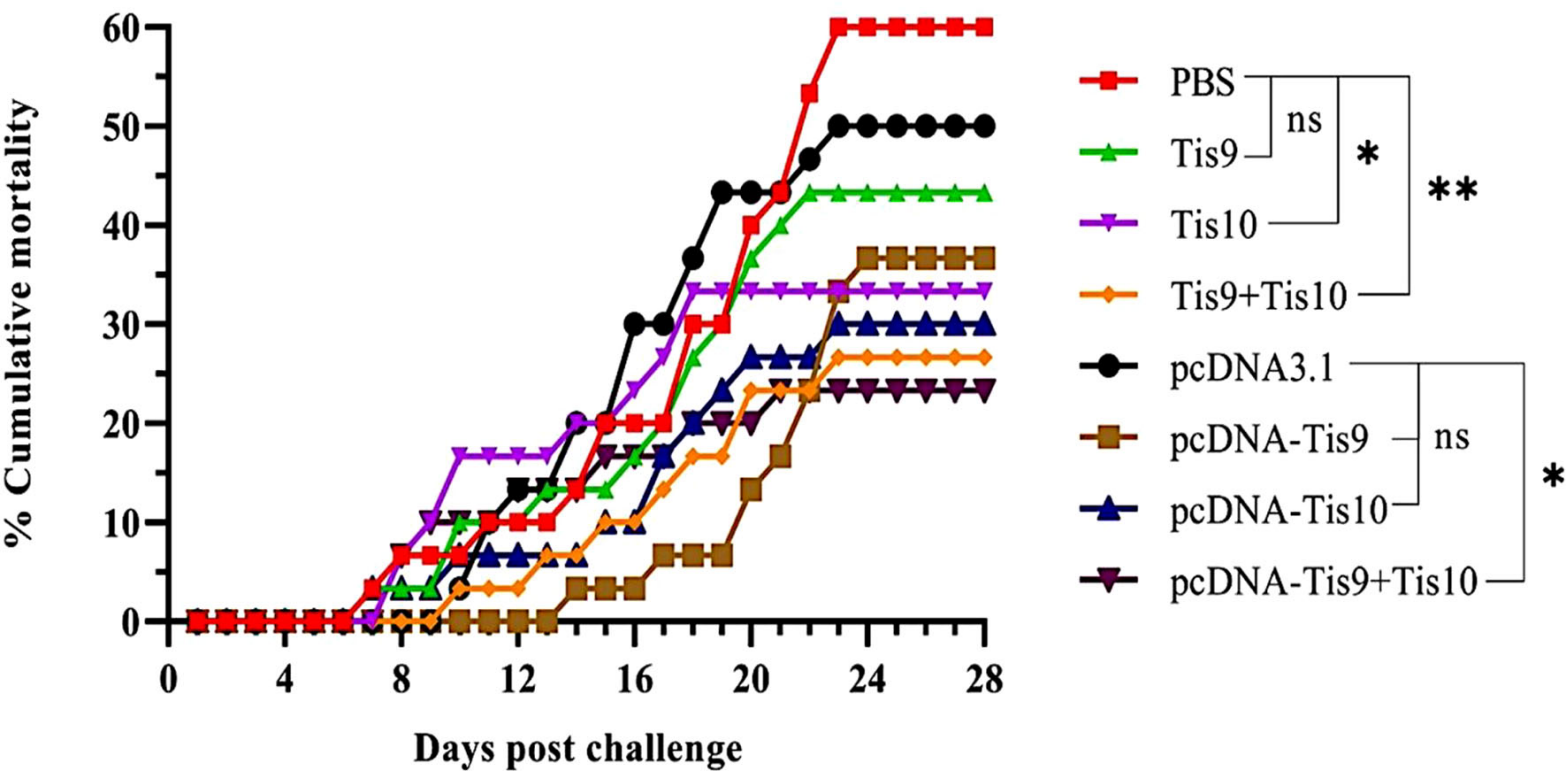
Cumulative mortality curves of the recombinant protein vaccines (Tis9, Tis10 and Tis9+Tis10 groups) and DNA vaccines (pcDNA-Tis9, pcDNA-Tis10 and pcDNA-Tis9+Tis10 groups) after challenge with 1 x 10^5^ TCID50 of TiLV. Differences among the groups were analyzed using one-way ANOVA and Tukey’s *post hoc* tests. Statistical significance is indicated by single asterisk (*, P < 0.05) or double asterisks (**, P < 0.01) or ns (not significant) compared to the PBS or pcDNA3.1 (negative control) groups at the termination day (day 28).

Mortality reached a peak in almost all fish groups during weeks 3 or 4 after the viral challenge. At the end of the experiment, Tis10 and Tis9 + Tis10 resulted in cumulative mortality rates of 33.33 ± 15.28% and 26.67 ± 5.77%, respectively and significantly lower than the PBS group at 60.00 ± 10.00% (*p* < 0.05) (**Figure 3**). For DNA vaccines, efficacy testing showed that cumulative mortalities reached 36.67 ± 5.77% and 30.00 ± 10.00% in pcDNA-Tis9 and pcDNA-Tis10, respectively and were not significantly different from the pcDNA3.1 group at 50.00 ± 00.00% (*p* > 0.05). However, in the pcDNA-Tis9 + Tis10 group, mortality was significantly lower than in the pcDNA3.1 group (23.33 ± 5.77%) (*p* < 0.05) (**Figure 3**).

The pcDNA-Tis10, pcDNA-Tis9 + Tis10 and Tis9 +Tis10 groups demonstrated similar RPS values at 50.00 ± 16.67%, 61.11 ± 9.62% and 55.56 ± 9.62%, respectively and not significantly different from the Tis10 and pcDNA-Tis9 groups at 44.45 ± 25.46% and 38.89 ± 9.62%, respectively. However, they were significantly higher than the Tis9 and pcDNA3.1 group, which showed 27.78 ± 9.62% and 16.67 ± 0.00%, respectively (**Table 3**).

**Table 3:** Cumulative mortality and RPS values of vaccinated fish at 28 days post TiLV challenge.

Group	Dose of vaccine (µg/fish)	Administration route	Number of fish challenged	Challenge dose (TCID_50_/fish)	No. of dead fish/total fish	Cumulative mortality (%)	RPS (%)
PBS	–	IP	10 (3 replicates)	1 × 10^5^	18/30	60.00 ± 10.00	–
pcDNA3.1	–	IM	10 (3 replicates)	1 × 10^5^	15/30	50.00 ± 00.00	16.67 ± 00.00^a^
Tis9	200 µg	IP	10 (3 replicates)	1 × 10^5^	13/30	43.33 ± 5.77	27.78 ± 9.62^a^
Tis10	200 µg	IP	10 (3 replicates)	1 × 10^5^	10/30	33.33 ± 15.28	44.45 ± 25.46^ab^
Tis9 + Tis10	200 µg	IP	10 (3 replicates)	1 × 10^5^	8/30	26.67 ± 5.77	55.56 ± 9.62^b^
pcDNA-Tis9	5 µg	IM	10 (3 replicates)	1 × 10^5^	11/30	36.67 ± 5.77	38.89 ± 9.62^ab^
pcDNA-Tis10	5 µg	IM	10 (3 replicates)	1 × 10^5^	9/30	30.00 ± 10.00	50.00 ± 16.67^b^
pcDNA-Tis9 + pcDNA-Tis10	5 µg	IM	10 (3 replicates)	1 × 10^5^	7/30	23.33 ± 5.77	61.11 ± 9.62^b^

Data are represented as means ± SDs (n = 3). Differences in RPS were analyzed using one-way ANOVA and Tukey’s post hoc tests, compared with the pcDNA3.1 (control group). Different letters indicate significant differences (P < 0.05). IP, intraperitoneal injection; IM, intramuscular injection.

### Antibody response of tilapia following immunization with Tis9 and Tis10 vaccines

**Figure 4:**
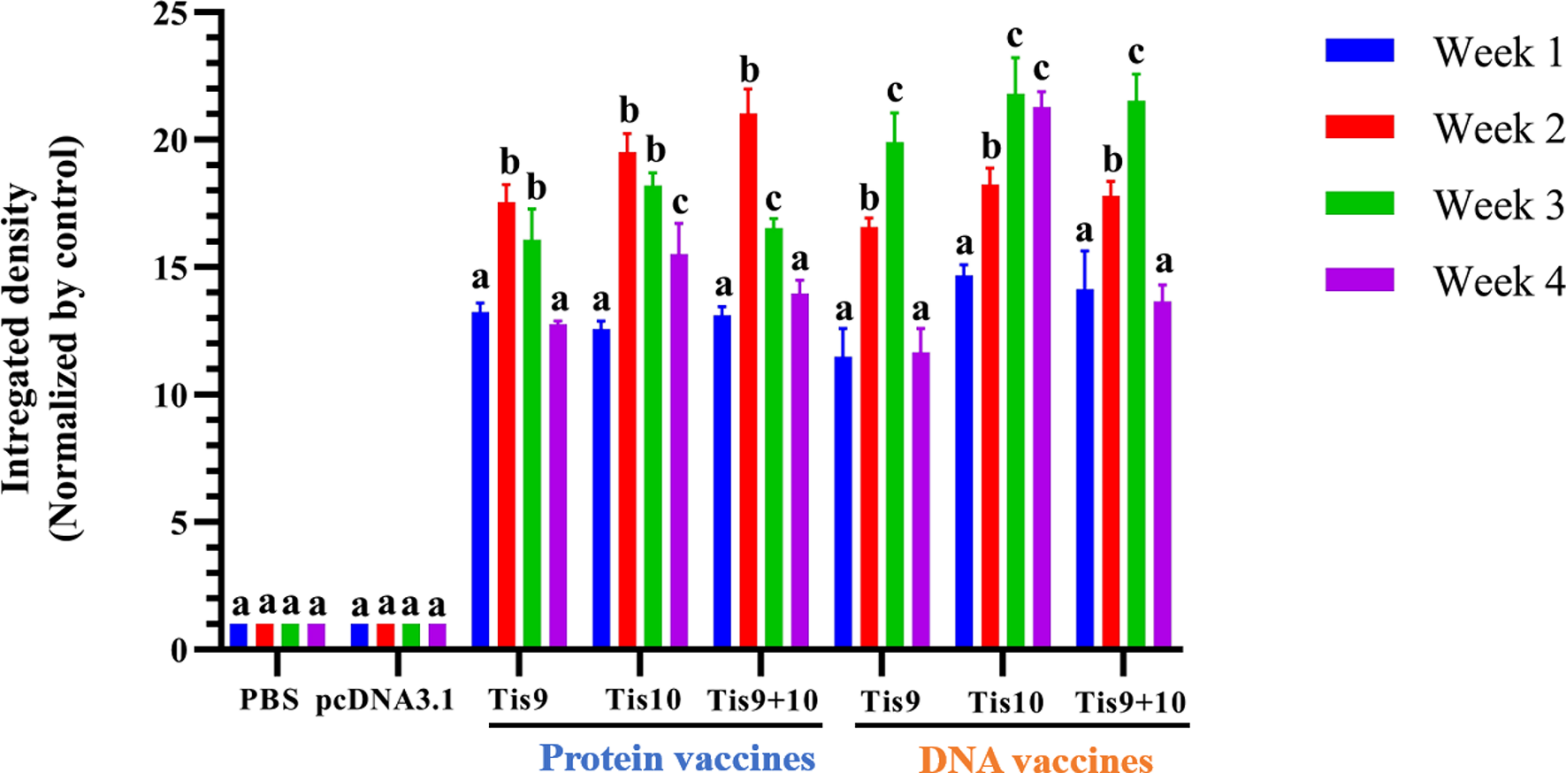
Dot blot analysis for identification of antibody production from vaccinated fish. Integrated densities from dot blot results were converted to values and normalized to the PBS or pcDNA3.1 negative control group using Image Lab software (Bio-Rad). Data are represented as means ± SD (n = 3). Different letters above the bars indicate significant differences within treatments (P < 0.05).

To determine the immune response after fish immunization with Tis9 and Tis10 recombinant protein and DNA vaccines, fish serum was collected for investigation by Dot Blot assay. Results demonstrated that fish immunized with the recombinant protein vaccines showed the highest antibody response during the 2^nd^ week, moderately dropped during the 3^rd^ and 4^th^ weeks. The pattern of immune response differed from the DNA vaccines, which gradually increased the production of fish antibodies from the 1^st^ to the 3^rd^ week, and then suddenly dropped during the 4^th^ week. Highest antibody levels were produced in the 2^nd^ or 3^rd^ week, with highest stimulation observed in the mixture of Tis9 and Tis10 vaccines. There were no positive signals from the PBS and pcDNA 3.1 control groups (**Figure 4**).

### Structural characterizations and B cell epitope prediction of Tis9 and Tis10

**Figure 5:**
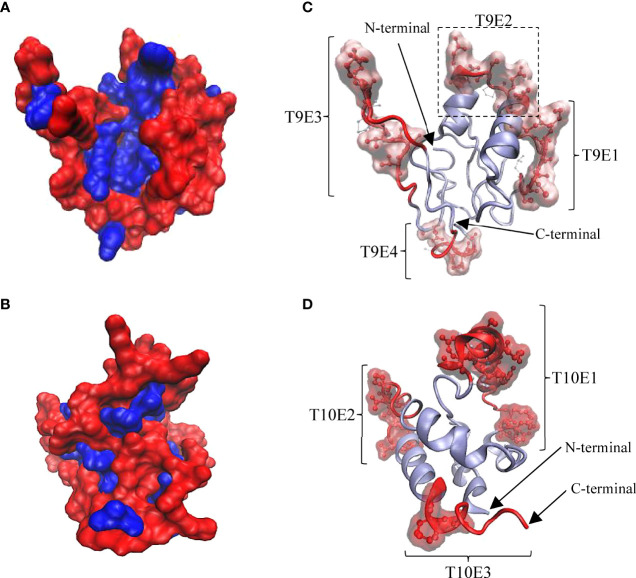
3D structure of Tis9 and Tis10 proteins showing their surface polarity, linear and conformational B cell epitope regions and relative proportions of amino acid residues in conformational epitopes. **(A)** Tis9 and **(B)** Tis10 models are shown in surface presentation, nonpolar residues (blue) buried in the core, whereas polar residues (red) tend to occur at the surface of proteins. (C and D) Predicted linear epitope and conformational epitope from the ElliPro results. Polar surface regions in **(A)** and **(B)** displayed linear epitopes (represented as red ribbon) and conformational epitopes (represented as red CPK along with surface structure on the Tis9 **(C)** and Tis10 **(D)** models. Their N-terminus and C-terminus are indicated by arrows.

To explain the antigenic properties of Tis9 and Tis10, bioinformatics analysis of B-cell epitope prediction in both proteins was conducted. The secondary (2D) structure and solvent accessibility were predicted by RaptorX Property server. As a result, Tis9 and Tis10 comprise 16% and 7% alpha-helix, 31% and 3% beta-strand and 51% and 89% coil, respectively (**Supplementary Tables 2**, **3**). Among the constituent amino acid residues of Tis9 and Tis10, predictions showed that 40% and 81% were predicted to be exposed, 24% and 6% medium exposed, while 35% and 12% were predicted to be buried, respectively. Further study of the 3D structure using the I-TASSER Server generated 3D models of Tis9 and Tis10 (**Figures 5A, B**). These models represented non-polar or hydrophobic residues that tended to be buried in the interior, whereas polar or hydrophilic residues exposed to solvent were located on the surface of the proteins.

The antigenicity of Tis9 and Tis10 proteins were predicted as 0.436% and 0.620%, respectively, at a 0.4% threshold for the viral model. ElliPro server was used to predict the B-cell epitopes in the refined 3D structure of Tis9 and Tis10 obtained from the I-TASSER Server. The linear and conformational epitopes were predicted based on the integration of antigenicity, flexibility and solvent accessibility of the protein structure (25). Using a cut-off minimum score ≥ 0.6 and maximum distance ≥ 5 angstroms, all predicted B-cell epitopes are listed in **Tables S2**, **S3**.

As shown in **Figure 5C**, in Tis9, 4 of the linear and conformational B cell epitope regions were predicted and found on the surface area, involving T9E1, T9E2, T9E3 and T9E4 epitopes. Also, 24 out of 27 conformational epitope residues were in linear epitope regions. Considering Tis10, 3 linear B cell epitope regions were predicted including T10E1, T10E2 and T10E3 epitopes, as shown in **Figure 5D**, while 24 out of 25 conformational epitope residues were located within linear epitope regions. Following the prediction of 2D and 3D structures, as well as the antigenicity and properties of all epitope residues, protein conformation and B cell epitope characteristics of Tis9 and Tis10 proteins were calculated, as summarized in **Table 4**. Results suggested that Tis10 was considerably more potent in activating the immune response than Tis9, with a greater number of total surface-exposed epitope residues in coil formation totaling 41.59%. These data indicated that Tis10 proteins may have more potential for boosting the tilapia immune response against TiLV infection compared to Tis9 proteins.

**Table 4:** Protein conformation and B cell epitope characteristics of Tis9 and Tis10 proteins.

Characteristic	Tis9	Tis10
Number of total epitope residues (% per total)	42 (35.29%)	49 (43.36%)
- Linear B cell epitope residues	39 (32.37%)	48 (42.48%)
- Conformational B cell epitope residues	27 (22.69%)	25 (22.12%)
Number of total surface exposed epitope residues (% per total)	37 (31.09%)	47 (41.59%)
Number of total surface exposed epitope residues in coil formation (% per total)	29 (24.37%)	47 (41.59%)

a Number of total epitope residues refers to non-redundant epitope residues united from both linear B cell epitope residues and conformational B cell epitope residues.

## Discussion

Tilapia lake virus (TiLV) is highly virulent and infects wild and farmed tilapia at all stages of development, especially the early stages (2, 8, 28–34). Outbreaks of TiLV have spread rapidly in over 16 countries on 4 continents in the last decade, associated with high tilapia morbidity and mortality (2, 35–41). To date, vaccination is the most sustainable and effective strategy for protection against fish viral diseases (6, 42–45) but there are currently no affordable and effective commercially available vaccines against TiLV (4, 41).

Recently, continuous cell lines from tilapia such as TiB (brain), TG (gill), TH (heart), OnlL (liver) and OnlB (brain) have been successfully established and showed susceptibility to TiLV (46–48). Tilapia kidney (TK-1) cell line is another interesting cell line for virus isolation and propagation. This cell line has previously been used to determine the cytotoxicity of secretory products from *Streptococcus agalactiae* (49, 50). However, the viral susceptibility of TK-1, especially to TiLV infection remains unexplored and could be used as a diagnostic tool for TiLV infection. In our study, the CPEs of TiLV infected TK-1 were clearly visible compared with mock TK-1, with heavy intracellular vacuolation and detachment observed at the late stage of TiLV infection. The CPEs of TiLV infected TK-1 differed from TiLV infected OnlL, which demonstrated cell shrinkage and aggregation, plaque formation and syncytial formation (49), while granularity and elongation of cells followed by rounding and destruction of the monolayer were observed in OnlB cells (48). CPEs in TiLV infected TK-1 were similar to those observed in TiLV infected E-11, TG and TH cells, with the appearance of swollen, rounded and cytoplasmic vacuoles clearly observed from day 5 post-inoculation, and the cells become vastly detached thereafter (28, 47). Differences in CPEs possibly depended on cell types. Furthermore, the susceptibility and ability of TK-1 cells were demonstrated by specific TiLV gene amplification and virus purification from TiLV infected TK-1 cells. Purified TiLV showed rounded, enveloped virions of 75-80 nm, with characteristics similar to the morphology of TiLV. Moreover, the purified virions showed infectivity both *in vitro* and *in vivo* (tilapia) (data not shown). These results revealed that the TK-1 cell line can serve as a valuable *in vitro* tool for the propagation and isolation of TiLV. This system enabled the determination of virus-host interactions and the efficacy of vaccine candidates with host immune responses.

Currently, the functions of TiLV proteins are not characterized and not predicted by bioinformatics analysis. Therefore, the production of recombinant Tis protein could be a tool to analyze their functions. The pET32a system was utilized to obtain a higher expression level of recombinant Tis proteins expressed with different induction conditions, including temperature and time. Most of the recombinant Tis proteins showed expression in all conditions but the expressions of Tis 1-8 proteins were very low compared with Tis9 and Tis10 proteins. The Tis gene or protein sequences were speculated not to correspond with our tested conditions as these proteins might be toxic to *E coil* cells. Several factors affect protein expression including *E. coli* host strains, expression vectors, gene or protein sequences and expression conditions (51–55). Our methods and results concurred with recent studies regarding the expression and purification of TiLV segments 5, 6 and 8. TiLV segment 8, encoding a VP20 protein, was produced by pET32a-VP20 contained within *E. coli* strain BL21 (DE3) cells induced with 1 mM IPTG at 37 °C, while the recombinant VP20 (rVP20) protein was expressed as an insoluble protein (6). Segment 5 (S5) and segment 6 (S6) TiLV proteins were produced using the recombinant pET15b-thioredoxin vector containing partial S5 (S5_27-343_, S5_27-172_, S5_196-272_) and S6 (S6_30-317_, S6_30-190_, and S6_200-317_) in *E. coli* strain BL21(DE3) cells induced with 0.1 mM IPTG at 16 °C. Only S5_196-272_ and S6_200-317_ were expressed as soluble and insoluble proteins, respectively (56). This study successfully optimized conditions (1 mM IPTG at 15°C for 16 hr, 30°C for 6 hr and 37°C for 4 hr) for expression of the recombinant pET32a-Tis9 as an insoluble protein and pET32a-Tis10 as a soluble protein. Subsequently, Tis9 and Tis10 proteins were successfully purified by affinity chromatography using a Ni-NTA column with native condition, which could be further used for functional assay. Tis9 and Tis10 were also subjected to further analysis regarding the highly conserved nucleotide level and amino acid level (52). Additionally, this study revealed the technical effort involved in large-scale production regarding purity and amount of Tis9 and Tis10 proteins. Both recombinant proteins showed increased size resulting from an additional 6× - His tag at the N-terminus for protein detection and purification purposes.

Currently, vaccines are known as the most effective method to prevent a wide range of diseases in fish (57, 58). However, few studies have reported on the development of a vaccine to prevent TiLV infection in tilapia. Initially, the traditional TiLV vaccine was produced, in which TiLV was attenuated by 17 and 20 subsequent passages (P17 and P20) based on cell culture (59). This vaccine provided over 50% of RPS values with dose of 1.2 × 10^7^ (P17) and 8.9 × 10^6^ (P20) TCID_50_ mL^−1^ without adjuvant. Recently, β-propiolactone-inactivated TiLV vaccine coupled with the adjuvant Montanide IMS 1312 VG and a booster vaccination (3^rd^ week) provided 85.7% RPS with a dose of 10^8^ TCID_50_ mL^−1^ (4). In another study, primary vaccination with pV-optiVP20 (Tis-8) DNA vaccine (no adjuvant) and a booster (3^rd^ week) with recombinant VP20 (rVP20) protein vaccine (adjuvant M402) provided 71.8% RPS (6). This study tested the protective efficacy of recombinant protein (adjuvant Montanide ISA 70 VG) and DNA vaccine (no adjuvant) of Tis9 and Tis10 with different vaccine formulations, including Tis9 or Tis10 alone and a mixture of Tis9 and Tis10. Although booster vaccination was not performed in our study, both vaccine types of the mixture of Tis9 and Tis10 provided relatively high levels of protection (55.56 - 61.11% RPS) in vaccinated fish. This suggests that differences in the type and formulation of vaccine, type of adjuvant, dose of vaccine and booster immunization may contribute to the level of protection achieved by the vaccine.

Evaluations of Tis9 and Tis10 recombinant protein and DNA vaccines were tested *in vivo* in red tilapia. Apart from the six different vaccine formulae, individual Tis vaccine, both recombinant protein and DNA vaccine showed limited efficacy with less than 50% RPS. Compared to previous protein and DNA vaccines studied by Zeng et al. (4), our results were similar to VP20 (Tis-8) vaccine for both protein (50% RPS) and DNA vaccine (52.5% RPS). However, all VP20 (Tis-8) vaccination required a booster (3^rd^ week) to obtain promising protection (6). Hence, the best vaccination program of VP20 (Tis-8) was the DNA prime-recombinant protein boost immunization (6). Vaccine formulation might alter vaccine efficiency including 1) antigenic protein, 2) adjuvant used (adjuvant M402 enhanced aluminum of VP20 (Tis-8) and Montanide of Tis9 and Tis10), and 3) concentration of vaccine content [VP20 (Tis-8): 40 μg protein or 5 μg DNA vaccine] and [Tis9/Tis10: 200 µg protein per fish or 5 μg DNA vaccine].

TiLV often affects small tilapia, especially during the transfer from hatchery to open river hanging cage or earthen pond, as small fish size presents vaccine limitations. The aim of this vaccine development was to test a single dose immunization. According to our result, a single dose of the mixture of [Tis9 + Tis10] recombinant protein vaccine (55.56 ± 9.62% RPS), or the mixture of [pcDNA-Tis9 + pcDNA-Tis10] DNA vaccine (61.11 ± 9.62% RPS) showed significant elevation of RPS of vaccinated tilapia after TiLV infection. The efficacy of single dose vaccination was found similar to VP20 (Tis-8) DNA prime-recombinant protein boost immunization (6). Vaccine efficacy might reflect the type of adjuvant, type of antigenic protein and concentration of vaccine content.

The type of vaccine also impacts immune stimulation and protection of tilapia from TiLV infection. From our evidence, DNA vaccine stimulated specific antibody titer more than recombinant protein vaccine but this elevated immunity was slower than recombinant protein vaccine by approximately a week. Highest antibody production was recorded in the pcDNA-Tis10 group, consistent with the efficacious of this vaccine. Interestingly, the sustained production of antibodies in the pcDNA-Tis10 group remained high, even after 4 weeks of vaccination. The time required for DNA vaccine processing until specific immunity activation, including DNA vaccine uptake, expression and post-translational processing to stimulate host immunity was within 1 week of the injection method. The increase in immunity between the 2^nd^ week and highest immunity in the 3^rd^ week might protect the fish from TiLV infection, especially when transferring tilapia fry from the hatchery to the grow-out stage in cages or ponds. Results suggested that a single vaccination of Tis9 and Tis10 protein and DNA vaccines without booster immunization prolonged the activation of immune response for up to 3 weeks. This administration of the vaccine has some advantages for farmers, such as reducing cost, labor and time needed to vaccinate the fish, as well as reducing the stress on the fish cause by multiple administration in the fish production cycle. These two models of vaccine types as recombinant protein and DNA vaccine could effectively reduce losses by TiLV from “Tilapia One Month Mortality Syndrome (TOMMS)”, which commonly occurs during the first month after fish transfer. In addition, recombinant protein and DNA vaccines are considered safer to administer than attenuated live vaccine since they cannot revert to virulence and no risk of viral replication and pathogenicity in the host (6, 56, 58). Nevertheless, this study is the development and testing of TiLV vaccines on laboratory scale. There are currently practical challenges in implementing of these vaccine candidates for industrial tilapia farming. Administration of vaccines by injection to small tilapia in large numbers can be complicated and inappropriate, which may cause adverse effects on the fish (10, 12). Therefore, TiLV vaccine must be developed to be more effective and conducive to use in tilapia farms. Currently, oral and immersion vaccination have been developed to prevent several infectious pathogens in fish. Both vaccination methods have some benefits of suitable and easy administration in different sizes of fish for large-scale farming, and they can be administered several times with low stress to the fish (10, 12, 59). Importantly, the development of new vaccines against TiLV infection requires the new knowledge and advances in molecular techniques and biotechnology in order to achieve the cost-effective, practical, and environmentally friendly vaccine (10, 56, 60).

For unidentified proteins, especially TiLV proteins, the criteria for selecting proteins for vaccine preparation do not relate to the protein function but should consider immunogenic properties and secondary structures of target proteins. With specific antibody production to protect against TiLV in tilapia, the synergistic effect of protein immunogenic properties might result from the combination of the number of B-cell epitopes, the alignment of amino acids in the epitope and their secondary structure. Selection of antigenic proteins or epitopes for rationale vaccine design should include 1) overall structure as > 40% coil structure (60–64), 2) > 50% amino acid residues exposed to the surface (61) and 3) a large number of total surface exposed B-cell epitope residues in coil formation (61).

In conclusion, TiLV segments (Tis) genes were amplified and expressed as recombinant proteins in bacterial expression systems. Of these, *in vivo* protectivity was observed in both recombinant protein and DNA vaccines of Tis9 and Tis10. Tis10 showed higher efficiency than Tis9, while the synergistic effect increased in the combination of Tis9 + Tis10. *In silico* analysis affirmed the antigenic properties of the Tis-protein, which correlated to the elevation of a specific antibody in tilapia. The DNA vaccine showed higher efficacy than the recombinant protein vaccine. Finally, with high conservation of Tis9 and Tis10 among reported isolates, this vaccine might provide broad-spectrum protection among different isolates. Taken together, Tis9 and Tis10 are promising candidates for the development of vaccines against TiLV infection in tilapia and could be applied for protection in large-scale tilapia farming

## Data Availability Statement

The datasets presented in this study can be found in online repositories. The names of the repository/repositories and accession number(s) can be found in the article/**Supplementary Material**.

## Ethics Statement

The animal study was reviewed and approved by National Research Council of Thailand (NRCT).

## Author Contributions

PC performed all experiments, analyzed the results, and wrote the manuscript. HD purified and prepared TiLV. ES and EW assisted in the protein purification. SS, RM and NA assisted in tilapia culture. IH prepared the TK-1 cell line. PC, HD, EW, NA, IH and SU revised the manuscript. SU conceived and designed the study and analyzed and interpreted all the results. All authors have reviewed and approved the final manuscript.

## Acknowledgments

This research was supported by KURDI FF(KU) 17.64. The authors would like to thank the Department of Biochemistry, Faculty of Science, Kasetsart University for financial support and use of laboratory facilities, the Department of Aquaculture, Faculty of Fisheries, Kasetsart University for laboratory facilities and resources and the Aquatic Animal Health Research Center, Charoen Pokphand Co. Ltd. for providing the experimental fish. PC gratefully acknowledges funding support from the Science Achievement Scholarship of Thailand (SAST) and the Japan International Collaboration Agency and Science and Technology Research Partnership for Sustainable Development (JICA-SATREPS). The authors would like to thank Miss Thararat Phurahong for assistance in plasmid preparation.

## Conflict of Interest

Author RM is employed by Charoen Pokphand Foods Public Co., Ltd.

The remaining authors declare that this research was conducted in the absence of any commercial or financial relationships that could be construed as a potential conflict of interest.

## Publisher’s Note

All claims expressed in this article are solely those of the authors and do not necessarily represent those of their affiliated organizations, or those of the publisher, the editors and the reviewers. Any product that may be evaluated in this article, or claim that may be made by its manufacturer, is not guaranteed or endorsed by the publisher.

## Supplementary Material

Supplementary Figure 1
**(A)** Moribund fish showed skin redness and congestion around the eye and head (black arrow), pale skin with hemorrhage and erosion (blue arrow) and fin rot (red arrow). **(B)** Moribund fish from each group were screened for TiLV infection using RT-PCR and specific PCR products approximately 500 bps. Lane M: DNA marker; Lane P: positive control; Lane N: negative control; B: brain; L: liver; S: spleen.

Supplementary Table 1 Primers used in this study.

Supplementary Table 2 Secondary structure (H, alpha-helix; E, beta-sheet; C, coil), solvent accessibility (B, buried; M, medium expose; E, expose), linear and conformational B-cell epitope (E, epitope) prediction of Tis9 protein.

Supplementary Table 3 Secondary structure (H, alpha-helix; E, beta-sheet; C, coil), solvent accessibility (B, buried; M, medium expose; E, expose), linear and conformational B-cell epitope (E, epitope) prediction of Tis10 protein.

## References

[1] Senapin S Shyam KU Meemetta W Rattanarojpong T Dong HT. Inapparent infection cases of tilapia lake virus (TiLV) in farmed tilapia. Aquaculture (2018) 487:51–5. doi: 10.1016/J.AQUACULTURE.2018.01.007

[2] Jansen MD Dong HT Mohan CV. Tilapia lake virus: a threat to the global tilapia industry? Rev Aquacult (2019) 11:725–39. doi: 10.1111/raq.12254

[3] Liamnimitr P Thammatorn W U-thoomporn S Tattiyapong P Surachetpong W. Non-lethal sampling for tilapia lake virus detection by RT-qPCR and cell culture. Aquaculture (2018) 486:75–80. doi: 10.1016/j.aquaculture.2017.12.015

[4] Zeng W Wang Y Hu H Wang Q Bergmann SM Wang Y. Cell culture-derived tilapia lake virus-inactivated vaccine containing montanide adjuvant provides high protection against viral challenge for tilapia. Vaccines (Basel) (2021) 9:1–15. doi: 10.3390/vaccines9020086 PMC7911875 33503930

[5] Mai TT Kayansamruaj P Taengphu S Senapin S Costa JZ del-Pozo J. Efficacy of heat-killed and formalin-killed vaccines against tilapia tilapinevirus in juvenile nile tilapia (Oreochromis niloticus). J Fish Dis (2021) 44:2097–109. doi: 10.1111/jfd.13523 PMC9291230 34477227

[6] Zeng W Wang Y Chen X Wang Q Bergmann SM Yang Y. Potency and efficacy of VP20-based vaccine against tilapia lake virus using different prime-boost vaccination regimens in tilapia. Aquaculture (2021) 539:736654(1-11). doi: 10.1016/j.aquaculture.2021.736654

[7] Acharya V Chakraborty HJ Rout AK Balabantaray S Behera BK Das BK. Structural characterization of open reading frame-encoded functional genes from tilapia lake virus (TiLV). Mol Biotechnol (2019) 61:945–57. doi: 10.1007/s12033-019-00217-y 31664705

[8] Bacharach E Mishra N Briese T Zody MC Kembou E Zamostiano R. Characterization of a novel orthomyxo-like virus causing mass die- offs of tilapia. mBio (2016), 7:1–7. doi: 10.1128/mBio.00431-16.Editor PMC4959514 27048802

[9] Mugimba KK Lamkhannat M Dubey S Mutoloki S Munang’andu HM Evensen Ø. Tilapia lake virus downplays innate immune responses during early stage of infection in nile tilapia (Oreochromis niloticus). Sci Rep (2020) 10:1–13. doi: 10.1038/s41598-020-73781-y 33230226 PMC7684318

[10] Mondal H Thomas J. A review on the recent advances and application of vaccines against fish pathogens in aquaculture. Aquacult Int (2022) 1–30. doi: 10.1007/s10499-022-00884-w PMC9059915 35528247

[11] Adams A. Progress, challenges and opportunities in fish vaccine development. Fish Shellfish Immunol (2019) 90:210–4. doi: 10.1016/j.fsi.2019.04.066 31039441

[12] Cain KD Sudheesh PS. Prospects and challenges of developing and commercializing immersion vaccines for aquaculture (2017). Available at: https://www.researchgate.net/publication/320112090.

[13] Bao P Sun X Liu Q Zhang Y Liu X. Synergistic effect of a combined live vibrio anguillarum and edwardsiella piscicida vaccine in turbot. Fish Shellfish Immunol (2019) 88:84–90. doi: 10.1016/j.fsi.2019.02.014 30763616

[14] Peng B Lin Xp Wang Sn Yang Mj Peng Xx Li H. Polyvalent protective immunogens identified from outer membrane proteins of vibrio parahaemolyticus and their induced innate immune response. Fish Shellfish Immunol (2018) 72:104–10. doi: 10.1016/j.fsi.2017.10.046 29107742

[15] Lueangyangyuen A Senapin S Dong HT Unajak S Wangkahart E Khunrae P. Expression and purification of S5196-272 and S6200-317 proteins from tilapia lake virus (TiLV) and their potential use as vaccines. Protein Expression Purification (2022) 190:106013. doi: 10.1016/j.pep.2021.106013 34752859

[16] Gautam A Tiwari A Malik YS. Bioinformatics applications in advancing animal virus research. Recent Adv Anim Virol (2019) 447–71. doi: 10.1007/978-981-13-9073-9_23

[17] Wang Y Wang E He Y Wang K Yang Q Wang J. Identification and screening of effective protective antigens for channel catfish against streptococcus iniae. Oncotarget (2017) 8:30793–804. doi: 10.18632/oncotarget.16475 PMC5458168 28415641

[18] Pumchan A Krobthong S Roytrakul S Sawatdichaikul O Kondo H Hirono I. Novel chimeric multiepitope vaccine for streptococcosis disease in Nile tilapia (Oreochromis niloticus Linn). Sci Rep (2020) 10:1–15. doi: 10.1038/s41598-019-57283-0 31953479 PMC6969146

[19] Delrue I Verzele D Madder A Nauwynck HJ. Inactivated virus vaccines from chemistry to prophylaxis: Merits, risks and challenges. Expert Rev Vaccines (2012) 11:695–719. doi: 10.1586/erv.12.38 22873127

[20] Wang S Li W Liu S Xu J. RaptorX-property: A web server for protein structure property prediction. Nucleic Acids Res (2016) 44:W430–5. doi: 10.1093/nar/gkw306 PMC4987890 27112573

[21] Yang J Yan R Roy A Xu D Poisson JZY. The I-TASSER suite: protein structure and function prediction. Nat Methods (2015) 12:7–8. doi: 10.1038/nmeth.3213 PMC4428668 25549265

[22] Heo L Park H Seok C. GalaxyRefine: Protein structure refinement driven by side-chain repacking. Nucleic Acids Res (2013) 41:W384–8. doi: 10.1093/nar/gkt458 PMC3692086 23737448

[23] Laskowski RA Rullmannn JA MacArthur MW Kaptein R TJM. AQUA and PROCHECK-NMR: programs for checking the quality of protein structures solved by NMR. J Biomol NMR (1996) 8:477–86. doi: 10.1007/BF00228148 9008363

[24] Doytchinova IA Flower DR. Vaxijen: a server for prediction of protective antigens, tumor antigens and subunit vaccines. BMC Bioinf (2007) 8:1–7. doi: 10.1186/1471-2105-8-4 PMC1780059 17207271

[25] Ponomarenko J Bui HH Li W Fusseder N Bourne PE Sette A. ElliPro: A new structure-based tool for the prediction of antibody epitopes. BMC Bioinf (2008) 9:1–8. doi: 10.1186/1471-2105-9-514 PMC2607291 19055730

[26] Humphrey W Dalke A Schulten K. VMD: visual molecular dynamics. J Mol Graph (1996) 14(33-38):27–38. doi: 10.1016/0263-7855(96)00018-5 8744570

[27] Thawornwattana Y Dong HT Phiwsaiya K Sangsuriya P Senapin S Aiewsakun P. Tilapia lake virus (TiLV): Genomic epidemiology and its early origin. Transboundary Emerging Dis (2020) 68(2):0–2. doi: 10.1111/tbed.13693 32578388

[28] Eyngor M Zamostiano R Kembou Tsofack JE Berkowitz A Bercovier H Tinman S. Identification of a novel RNA virus lethal to tilapia. J Clin Microbiol (2014) 52:4137–46. doi: 10.1128/JCM.00827-14 PMC4313277 25232154

[29] Amal MNANA Koh CBB Nurliyana M Suhaiba M Nor-Amalina Z Santha S. A case of natural co-infection of tilapia lake virus and aeromonas veronii in a Malaysian red hybrid tilapia (Oreochromis niloticus × o. mossambicus) farm experiencing high mortality. Aquaculture (2018) 485:12–6. doi: 10.1016/j.aquaculture.2017.11.019

[30] Dong HT Ataguba GA Khunrae P Rattanarojpong T Senapin S. Evidence of TiLV infection in tilapia hatcheries in Thailand from 2012 to 2017 reveals probable global spread of the disease. Aquaculture (2017) 479:579–83. doi: 10.1016/j.aquaculture.2017.06.035

[31] Jaemwimol P Rawiwan P Tattiyapong P Saengnual P Kamlangdee A Surachetpong W. Susceptibility of important warm water fish species to tilapia lake virus (TiLV) infection. Aquaculture (2018) 497:462–8. doi: 10.1016/j.aquaculture.2018.08.028

[32] Mugimba KK Chengula AA Wamala S Mwega ED Kasanga CJ Mdegela DKBRH. Detection of tilapia lake virus (TiLV) infection by PCR in farmed and wild Nile tilapia ( oreochromis niloticus ) from lake Victoria. J Fish Dis (2018) 1–9. doi: 10.1111/jfd.12790 29473649

[33] Mugimba KK Tal S Dubey S Mutoloki S Dishon A Evensen A. Gray (Oreochromis niloticus X o. aureus) and red (Oreochromis spp.) tilapia show equal susceptibility and proinflammatory cytokine responses to experimental tilapia lake virus infection. Viruses (2019) 11:893(1-10). doi: 10.3390/v11100893 PMC6832934 31554184

[34] Tattiyapong P Dachavichitlead W Surachetpong W. Experimental infection of tilapia lake virus (TiLV) in Nile tilapia (Oreochromis niloticus) and red tilapia (Oreochromis spp.). Vet Microbiol (2017) 207:170–7. doi: 10.1016/j.vetmic.2017.06.014 28757020

[35] Surachetpong W Janetanakit T Nonthabenjawan N Tattiyapong P Sirikanchana K Amonsin A. Outbreaks of tilapia lake virus infection, Thailand, 2015–2016. Emerging Infect Dis (2017) 23(6):1031–3. doi: 10.3201/eid2306.161278 PMC5443430 28518020

[36] Del-Pozo J Mishra N Kabuusu R Cheetham S Eldar A Bacharach E. Syncytial hepatitis of tilapia (Oreochromis niloticus l.) is associated with orthomyxovirus-like virions in hepatocytes. Vet Pathol (2017) 54:164–70. doi: 10.1177/0300985816658100 27511312

[37] Mugimba KK Chengula AA Wamala S Mwega ED Kasanga CJ Byarugaba DK. Detection of tilapia lake virus (TiLV) infection by PCR in farmed and wild Nile tilapia (Oreochromis niloticus) from lake Victoria. J Fish Dis (2018) 41:1181–9. doi: 10.1111/jfd.12790 29473649

[38] Nicholson P Fathi MA Fischer A Mohan C Schieck E Mishra N. Detection of tilapia lake virus in Egyptian fish farms experiencing high mortalities in 2015. J Fish Dis (2017) 40:1925–8. doi: 10.1111/jfd.12650 28590067

[39] FAO. The state of world fisheries and aquaculture, 2018: Meeting the sustainable development goals (Rome). Fish Oceanogr (2018) 29:227–37. doi: 10.1111/fog.12466

[40] Aich N Paul A Choudhury TG Saha H. Tilapia lake virus (TiLV) disease: Current status of understanding. Aquacult Fish (2021) 7:7–17. doi: 10.1016/j.aaf.2021.04.007

[41] Surachetpong W Roy SRK Nicholson P. Tilapia lake virus: The story so far. J Fish Dis (2020) 43:1115–32. doi: 10.1111/jfd.13237 32829488

[42] Zeng W Wang Q Wang Y Zhao C Li Y Shi C. Immunogenicity of a cell culture-derived inactivated vaccine against a common virulent isolate of grass carp reovirus. Fish Shellfish Immunol (2016) 54:473–80. doi: 10.1016/j.fsi.2016.04.133 27142935

[43] Hjeltnes B Bornø G Jansen MD Haukaas A Walde C. The health situation in Norwegian aquaculture 2016. In: Norwegian Veterinary institute report series; Norwegian veterinary institute. Oslo, Norway: Norwegian Veterinary Institute (2017).

[44] Jee YH Mun-Gyeong K Yu JK Sung HJ Myoung AP Maeng HS. Montanide IMS 1312 VG adjuvant enhances the efficacy of immersion vaccine of inactivated viral hemorrhagic septicemia virus (VHSV) in olive flounder, paralichthys olivaceus. Fish Shellfish Immunol (2017) 60:420–5. doi: 10.1016/j.fsi.2016.12.011 27965163

[45] Matsuura Y Terashima S Takano T Matsuyama T. Current status of fish vaccines in Japan. Fish Shellfish Immunol (2019) 95:236–47. doi: 10.1016/j.fsi.2019.09.031 31586679

[46] Wang Y Wang Q Zeng W Yin J Li Y Ren Y. Establishment and characterization of a cell line from tilapia brain for detection of tilapia lake virus. J Fish Dis (2018) 41:1803–9. doi: 10.1111/jfd.12889 30320411

[47] Nanthini R Abdul Majeed S Vimal S Taju G Sivakumar S Santhosh Kumar S. *In vitro* propagation of tilapia lake virus in cell lines developed from oreochromis mossambicus. J Fish Dis (2019) 42:1543–52. doi: 10.1111/jfd.13075 31515819

[48] Thangaraj RS Ravi C Kumar R Dharmaratnam A Valaparambil Saidmuhammed B Pradhan PK. Derivation of two tilapia (Oreochromis niloticus) cell lines for efficient propagation of tilapia lake virus (TiLV). Aquaculture (2018) 492:206–14. doi: 10.1016/J.AQUACULTURE.2018.04.012

[49] Palang I Hirono I Senapin S Sirimanapong W Withyachumnarnkul B Vanichviriyakit R. Cytotoxicity of streptococcus agalactiae secretory protein on tilapia cultured cells. J Fish Dis (2020) 43:1229–36. doi: 10.1111/jfd.13230 32974952

[50] Chen SN Ueno Y Wen SC Kou & G H. Establishment of a cell line from kidney of tilapia, in: Establishment of a cell line from kidney of tilapia bulletin- European association of fish pathologists (1983). Dayeh. Available at: https://eafp.org/download/1983-Volume3/Issue%201/03_1%20p1.PDF (Accessed June 29, 2018).

[51] Liu ZQ Yang PC. Construction of pET-32 α (+) vector for protein expression and purification. North Am J Med Sci (2012) 4:651–5. doi: 10.4103/1947-2714.104318 PMC3530323 23272309

[52] Chaput DL Bass D Alam MM Hasan N Stentiford GD van Aerle R. The segment matters: Probable reassortment of tilapia lake virus (TiLV) complicates phylogenetic analysis and inference of geographical origin of new isolate from Bangladesh. Viruses (2020) 12:1–17. doi: 10.3390/v12030258 PMC7150994 32120863

[53] Evensen O. Development of fish vaccines: Focusing on methods. In: Adams A, editor. Fish vaccines. Springer, Basel: Springer. (2016) p. 53–74. doi: 10.1007/978-3-0348-0980-1_3

[54] Francis DM Page R. Strategies to optimize protein expression in e. coli. Curr Protoc Protein Sci (2010) 5(1). doi: 10.1002/0471140864.ps0524s61 PMC7162232 20814932

[55] Rosano GL Ceccarelli EA. Recombinant protein expression in escherichia coli: advances and challenges. Front Microbiol (2014) 5:172. doi: 10.3389/fmicb.2014.00172 24860555 PMC4029002

[56] Ma J Bruce TJ Jones EM Cain KD. A review of fish vaccine development strategies: Conventional methods and modern biotechnological approaches. Microorganisms (2019) 7:569(1–18). doi: 10.3390/microorganisms7110569 PMC6920890 31744151

[57] Bacharach E Eldar A. Tilapia lake virus vaccines. US Patent Application Publication (2016). Available at: https://patents.google.com/patent/US20160354458A1/en.

[58] Celene SM Elizabeth LR Edith RA Gary G. Viral vaccines for bony fish: past, present and future. Expert Rev Vaccines (2014) 12:567–78. doi: org/10.1586/erv.13.38 10.1586/erv.13.38 23659303

[59] Reyes M Ramírez C Ñancucheo I Villegas R Schaffeld G Kriman L. A novel “in-feed” delivery platform applied for oral DNA vaccination against IPNV enables high protection in Atlantic salmon (Salmon salar). Vaccine (2017) 35:626–32. doi: 10.1016/j.vaccine.2016.12.013 28012776

[60] Adams A. Progress, challenges and opportunities in fish vaccine development. Fish Shellfish Immunol (2019) 90:210–4. doi: 10.1016/j.fsi.2019.04.066 31039441

[61] Azim KF Lasker T Akter R Hia MM Bhuiyan OF Hasan M. Combination of highly antigenic nucleoproteins to inaugurate a cross-reactive next generation vaccine candidate against arenaviridae family. Heliyon (2021) 7:e07022. doi: 10.1016/j.heliyon 34041391 PMC8144012

[62] Ezediuno LO Onile OS Oladipo EK Majolagbe ON Jimah EM Senbadejo TY. Designing multi-epitope subunit vaccine for ocular trachoma infection using chlamydia trachomatis polymorphic membrane proteins G. Inf Med Unlocked (2021) 26:100764. doi: 10.1016/j.imu.2021.100764

[63] Shrivastava N Verma A Dash PK. Identification of functional epitopes of structural proteins and in-silico designing of dual acting multiepitope anti-tick vaccine against emerging Crimean-Congo hemorrhagic fever virus. Eur J Pharm Sci (2020) 151:105396. doi: 10.1016/j.ejps.2020.105396 32479862

[64] Soltan MA Elbassiouny N Gamal H Elkaeed EB Eid RA Eldeen MA. In silico prediction of a multitope vaccine against moraxella catarrhalis: Reverse vaccinology and immunoinformatics. Vaccines (Basel) (2021) 9:1–13. doi: 10.3390/vaccines9060669s PMC8234879 34207238

