# Supplementary material for: Combining segments 9 and 10 in DNA and recombinant protein vaccines conferred superior protection against tilapia lake virus in hybrid red tilapia (oreochromis sp.) compared to single segment vaccines
Source: Front Immunol. 2022 Jul 25;13:935480. doi: 10.3389/fimmu.2022.935480 (PMC9359061; doi:10.3389/fimmu.2022.935480)
Supplement: Supplementary Figure 1 — (A) Moribund fish showed skin redness and congestion around the eye and head (black arrow), pale skin with hemorrhage and erosion (blue arrow) and fin rot (red arrow). (B) Moribund fish from each group were screened for TiLV infection using RT-PCR and specific PCR products approximately 500 bps. Lane M: DNA marker; Lane P: positive control; Lane N: negative control; B: brain; L: liver; S: spleen. [file DataSheet_1.pdf]

Supplementary Information:

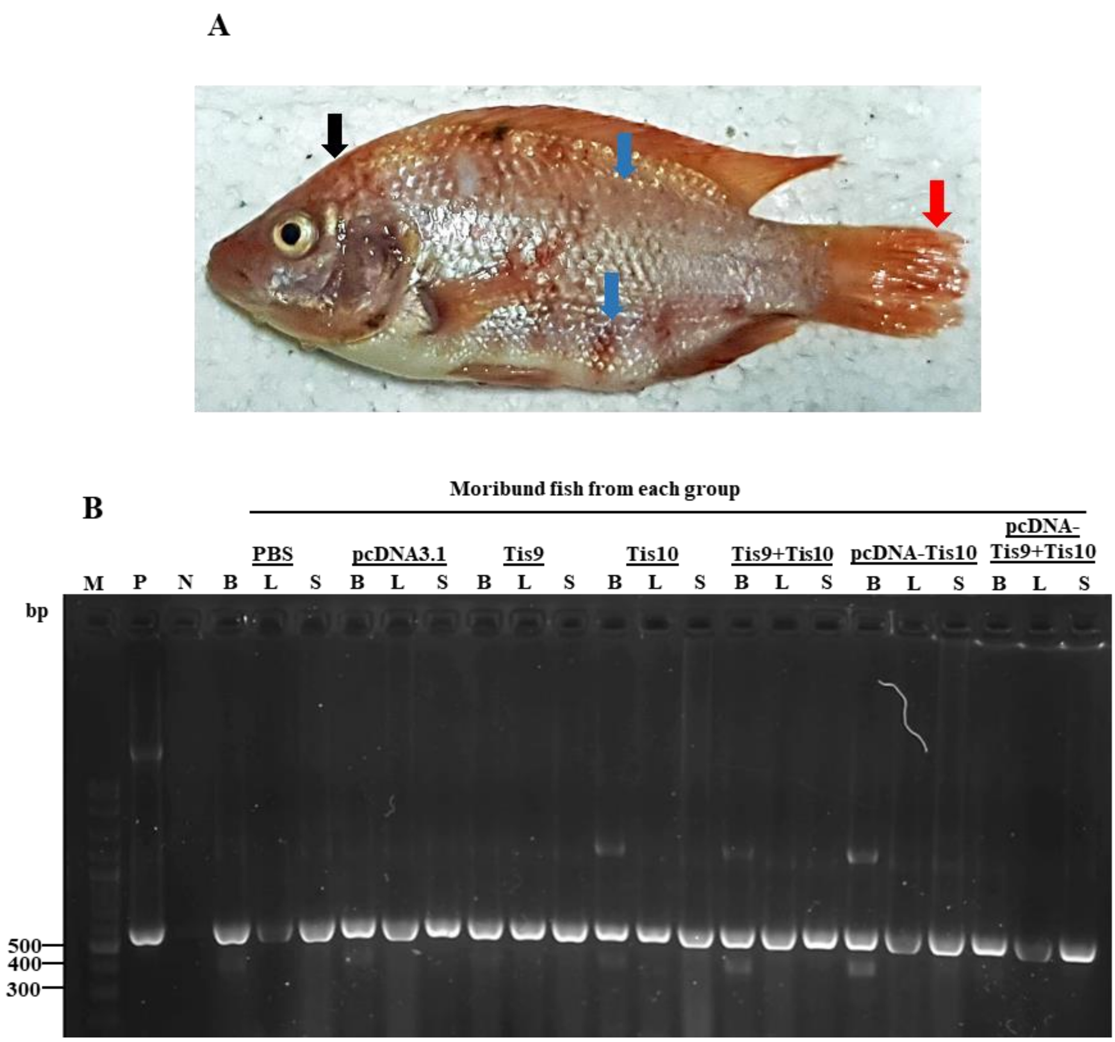

Supplementary Figure 1. (A) Moribund fish showed skin redness and congestion around the eye and head (black arrow), pale skin with hemorrhage and erosion (blue arrow) and fin rot (red arrow). (B) Moribund fish from each group were screened for TiLV infection using RT-PCR and specific PCR products approximately 500 bps. Lane M: DNA marker; Lane P: positive control; Lane N: negative control; B: brain; L: liver; S: spleen.
