# Supplementary material for: Combining segments 9 and 10 in DNA and recombinant protein vaccines conferred superior protection against tilapia lake virus in hybrid red tilapia (oreochromis sp.) compared to single segment vaccines
Source: Front Immunol. 2022 Jul 25;13:935480. doi: 10.3389/fimmu.2022.935480 (PMC9359061; doi:10.3389/fimmu.2022.935480)
Supplement: Supplementary Table 1 — Primers used in this study. [file Table_1.pdf]

Supplementary Information:

Supplementary Table 1. Primers used in this study.

TiLV segment	GenBank accession number	Primer name	Sequence (5'- 3')
1	KX631921	SpecificTiLV-F	CAGGGAGAAAGCCCACTGAG
		SpecificTiLV-R	CTCCAATTGACCCCATGCCT
1	KX631921	Tis1-F	GGATCCATGTGGGCATTTCAAGAAGGAGT (*BamHI*)
		Tis1-R	AAGCTTTTAGCACCCAGCGGTGGGCT (*HidIII*)
2	KX631922	Tis2-F	GAGCTCATGAGTCAGTTTGGGAAAT (*SacI*)
		Tis2-R	GCGGCCGCCTACTGATTTAGATCCATATT (*NotI*)
3	KX631923	Tis3-F	GGATCCATGGACTCGCGGTTTGCAC (*BamHI*)
		Tis3-R	AAGCTTTTATCTCGCAAATGGGTGTACTGTC (*HidIII*)
4	KX631924	Tis4-F	GGATCCATGAAAATGGTGAGAACTACAAAG (*BamHI*)
		Tis4-R	CTCGAGCTATCTCCCAACAGCCCCTGC (*XhoI*)
5	KX631925	Tis5-F	GCGGATTCATGTTTCTCTTATCTCAG (*BamHI*)
		Tis5-R	GGTCTAGATCAAGATAATGGAAGCAG (*XbaI*)
6	KX631927	Tis6-F	GAATTCATGCATTTTTATCTACAGGATTA (*EcoRI*)
		Tis6-R	GGCTCAGATCACATGTATTTATTG (*XbaI*)

Supplementary Table 1 (Continued)

TiLV segment	GenBank accession number	Primer name	Sequence (5’- 3’)
7	KX631927	Tis7-F	GGATCCATGTCCTACAAGATTGGTGAGCTTG (*BamHI*)
		Tis7-R	AAGCTTTTAGAGTTCAAACGTGATTCCTTTAGG (*HidIII*)
8	KX631928	Tis8-F	GGTACCATGGCTCAAATCCCAACAC (*KpnI*)
		Tis8-R	GCGGCCGCTCATTTAAGCATTTCACGG (*NotI*)
9	KX631929	Tis9-F	GAATTCATGTTGGTGATGTCACGATGG (*EcoRI*)
		Tis9-R	GCGGCCGCTCATAAAGTTCTATCGCCAGCCATG (*NotI*)
9	KX631929	TiLV-S9-F	GGAAGCTTATGGTGATGTCACGATG (*HidIII*)
		TiLV-S9-R	GGCTCGAGTCATAAAGTTCTATCGC (*XhoI*)
10	KX631930	Tis10-F	GGATCCATGAGTGTGGCAGATTATTTGTC (*BamHI*)
		Tis10-R	AAGCTTCTAAGACTCCACGTCAAGAG (*HidIII*)
10	KX631930	TiLV-S10-F	GCGGATCCATGAGTGTGGCAGAT (*BamHI*)
		TiLV-S10-R	GGTCTAGACTAAGACTGCACGTC (*XbaI*)
