# Supplementary material for: Combining segments 9 and 10 in DNA and recombinant protein vaccines conferred superior protection against tilapia lake virus in hybrid red tilapia (oreochromis sp.) compared to single segment vaccines
Source: Front Immunol. 2022 Jul 25;13:935480. doi: 10.3389/fimmu.2022.935480 (PMC9359061; doi:10.3389/fimmu.2022.935480)
Supplement: Supplementary Table 2 — Secondary structure (H, alpha-helix; E, beta-sheet; C, coil), solvent accessibility (B, buried; M, medium expose; E, expose), linear and conformational B-cell epitope (E, epitope) prediction of Tis9 protein. [file Table_2.pdf]

Supplementary Information:

Supplementary Table 2. Secondary structure (H, alpha-helix; E, beta-sheet; C, coil), solvent accessibility (B, buried; M, medium expose; E, expose), linear and conformational B-cell epitope (E, epitope) prediction of Tis9 protein.

Residue	Amino acid	Secondary structure	Solvent accessibility	Linear B-cell epitope	Conformational B-cell epitope
1	M	C	E	-	-
2	L	C	E	-	-
3	V	E	E	-	-
4	M	E	E	-	-
5	S	E	E	-	-
6	R	E	E	-	-
7	W	E	M	-	-
8	I	E	B	-	-
9	E	C	E	-	-
10	N	C	E	-	-
11	T	C	E	-	-
12	D	C	E	-	-
13	S	C	E	-	-
14	V	E	B	-	-
15	S	E	M	-	-
16	V	E	B	-	-
17	I	E	M	-	-
18	L	E	B	-	-
19	T	E	M	-	-
20	E	E	E	-	-
21	M	C	E	-	-

Supplementary Table 2 (Continued)

Residue	Amino acid	Secondary structure	Solvent accessibility	Linear B-cell epitope	Conformational B-cell epitope
22	G	C	E	-	-
23	R	C	E	-	-
24	S	C	E	-	-
25	Y	E	M	-	-
26	V	E	B	-	-
27	T	E	M	-	-
28	L	E	B	-	-
29	C	E	B	-	-
30	H	E	E	-	-
31	Y	C	M	-	-
32	P	C	M	-	-
33	P	C	E	-	-
34	W	C	B	-	-
35	S	C	M	-	-
36	L	C	B	-	E
37	C	C	B	E	E
38	C	C	B	E	E
39	G	C	E	E	E
40	G	C	E	E	E
41	K	C	E	E	E
42	T	C	E	E	-
43	S	C	E	E	-
44	Q	C	E	E	-
45	P	C	E	E	-
46	G	C	M	E	-

Supplementary Table 2 (Continued)

Residue	Amino acid	Secondary structure	Solvent accessibility	Linear B-cell epitope	Conformational B-cell epitope
47	Q	E	M	-	-
48	V	E	B	-	-
49	W	E	B	-	-
50	I	E	B	-	-
51	L	E	B	-	-
52	D	E	M	-	-
53	R	C	M	-	-
54	K	C	E	-	-
55	H	E	M	-	-
56	C	E	B	-	-
57	L	E	B	-	-
58	E	E	E	-	-
59	C	C	B	-	-
60	S	C	E	-	-
61	E	C	E	-	-
62	I	H	B	-	-
63	S	H	M	-	-
64	E	H	E	-	E
65	L	H	B	-	-
66	M	C	M	-	-
67	T	C	E	E	E
68	L	C	M	E	-
69	G	C	E	E	E
70	Q	C	E	E	E
71	P	C	E	E	E
72	T	C	E	E	E

Supplementary Table 2 (Continued)

Residue	Amino acid	Secondary structure	Solvent accessibility	Linear B-cell epitope	Conformational B-cell epitope
73	W	H	B	E	-
74	Q	H	E	E	E
75	E	H	E	E	E
76	S	H	B	-	-
77	A	H	B	-	-
78	M	H	M	-	-
79	V	H	B	-	-
80	I	H	B	-	-
81	G	H	B	-	-
82	S	H	E	-	-
83	W	H	B	-	-
84	V	H	B	-	-
85	Q	H	M	-	-
86	V	H	B	-	-
87	V	H	B	-	-
88	T	H	M	-	-
89	E	C	E	-	E
90	H	C	E	-	-
91	G	C	B	-	-
92	I	E	B	-	-
93	L	E	B	E	-
94	H	E	M	E	-
95	T	C	M	E	E
96	S	C	E	E	E
97	T	C	E	E	E
98	S	C	E	E	E

Supplementary Table 2 (Continued)

Residue	Amino acid	Secondary structure	Solvent accessibility	Linear B-cell epitope	Conformational B-cell epitope
99	E	C	E	E	-
100	R	C	E	E	E
101	Q	C	E	E	E
102	A	C	E	E	E
103	P	C	E	E	E
104	R	C	E	E	-
105	R	E	E	E	-
106	V	E	B	E	-
107	M	E	M	-	-
108	W	E	B	-	-
109	R	E	M	-	-
110	I	E	B	-	-
111	S	C	M	-	-
112	D	C	E	E	-
113	M	C	M	E	-
114	A	C	E	E	E
115	G	C	E	E	E
116	D	C	E	E	E
117	R	C	E	E	E
118	T	C	E	-	-
119	L	C	E	-	-
