# Supplementary material for: Combining segments 9 and 10 in DNA and recombinant protein vaccines conferred superior protection against tilapia lake virus in hybrid red tilapia (oreochromis sp.) compared to single segment vaccines
Source: Front Immunol. 2022 Jul 25;13:935480. doi: 10.3389/fimmu.2022.935480 (PMC9359061; doi:10.3389/fimmu.2022.935480)
Supplement: Supplementary Table 3 — Secondary structure (H, alpha-helix; E, beta-sheet; C, coil), solvent accessibility (B, buried; M, medium expose; E, expose), linear and conformational B-cell epitope (E, epitope) prediction of Tis10 protein. [file Table_3.pdf]

Supplementary Information:

Supplementary Table 3. Secondary structure (H, alpha-helix; E, beta-sheet; C, coil), solvent accessibility (B, buried; M, medium expose; E, expose), linear and conformational B-cell epitope (E, epitope) prediction of Tis10 protein.

Residue	Amino acid	Secondary structure	Solvent accessibility	Linear B-cell epitope	Conformational B-cell epitope
1	M	C	E	-	-
2	S	C	E	-	-
3	V	H	E	-	-
4	A	H	E	-	-
5	D	H	E	-	-
6	Y	H	E	-	-
7	L	C	E	-	-
8	S	C	E	-	-
9	S	C	E	-	-
10	D	C	E	-	-
11	S	C	E	-	-
12	D	C	E	-	-
13	S	C	E	-	-
14	E	H	E	-	-
15	A	H	E	-	-
16	E	H	E	-	-
17	S	H	E	-	-
18	S	C	E	-	-
19	G	C	E	-	-
20	C	C	E	-	-
21	L	E	E	-	-

Supplementary Table 3 (Continued)

Residue	Amino acid	Secondary structure	Solvent accessibility	Linear B-cell epitope	Conformational B-cell epitope
22	V	E	M	-	-
23	L	E	B	-	-
24	R	E	M	-	-
25	S	H	B	-	-
26	R	H	E	-	-
27	K	H	E	-	-
28	I	H	M	-	E
29	R	H	E	E	E
30	K	H	E	E	-
31	G	H	E	E	E
32	K	H	E	E	-
33	K	H	E	E	-
34	A	H	E	E	E
35	A	H	E	E	E
36	S	H	E	E	E
37	K	H	E	E	E
38	K	H	E	E	E
39	R	H	E	E	-
40	S	C	E	E	E
41	W	C	B	E	-
42	K	C	E	E	E
43	N	C	E	E	E
44	E	C	E	E	E
45	R	C	E	E	-
46	Y	C	E	E	-

Supplementary Table 3 (Continued)

Residue	Amino acid	Secondary structure	Solvent accessibility	Linear B-cell epitope	Conformational B-cell epitope
47	G	C	E	E	-
48	A	C	E	E	-
49	D	C	E	E	-
50	E	C	E	E	-
51	R	C	E	E	E
52	G	C	E	E	E
53	E	C	E	E	E
54	D	C	E	E	E
55	N	C	E	E	-
56	I	C	B	-	-
57	E	C	E	-	-
58	W	C	M	-	-
59	G	C	E	-	-
60	D	C	E	-	-
61	E	C	E	-	-
62	V	C	M	-	-
63	D	C	E	-	-
64	L	C	B	-	-
65	E	C	E	-	-
66	M	C	M	-	-
67	D	C	E	-	-
68	D	C	E	-	-
69	C	C	M	-	-
70	D	C	E	-	-
71	S	C	E	-	-
72	A	C	E	-	-

Supplementary Table 3 (Continued)

Residue	Amino acid	Secondary structure	Solvent accessibility	Linear B-cell epitope	Conformational B-cell epitope
73	I	C	B	-	-
74	P	C	E	-	-
75	E	C	E	-	-
76	W	C	B	-	-
77	A	C	M	-	-
78	R	C	E	-	-
79	V	C	M	-	-
80	D	C	E	-	-
81	F	C	B	E	-
82	N	C	E	E	-
83	P	C	E	E	-
84	K	C	E	E	E
85	N	C	E	E	E
86	R	C	E	E	-
87	R	C	E	E	-
88	D	C	E	E	E
89	R	C	E	E	E
90	E	C	E	E	-
91	D	C	E	E	E
92	D	C	E	E	E
93	G	C	E	-	-
94	Q	C	E	-	-
95	S	C	E	-	-
96	D	C	E	-	-
97	L	H	E	-	-
98	S	H	E	-	-

Supplementary Table 3 (Continued)

Residue	Amino acid	Secondary structure	Solvent accessibility	Linear B-cell epitope	Conformational B-cell epitope
99	R	H	E	-	-
100	F	H	E	-	-
101	S	H	E	-	-
102	E	H	E	-	-
103	D	H	E	-	-
104	F	H	E	-	-
105	G	C	E	E	E
106	K	C	E	E	E
107	K	C	E	E	E
108	S	C	E	E	-
109	L	C	E	E	-
110	D	C	E	E	-
111	V	C	E	E	-
112	Q	C	E	E	-
113	S	C	E	E	-
